# Prescription Audit in Outpatient Pharmacy of a Tertiary Care Referral Hospital in Haryana Using World Health Organization/International Network of Rational Use of Drugs (WHO/INRUD) Core Prescribing Indicators: A Step Towards Refining Drug Use and Patient Care

**DOI:** 10.3390/pharmacy13020048

**Published:** 2025-03-25

**Authors:** Nikhil Verma, Shanmugapriya Vinayagam, Niti Mittal, Rakesh Mittal, Neeraj Bansal

**Affiliations:** 1 Pt. B.D. Sharma PGIMS, Rohtak 124001, Haryana, India; vermanikhil512@gmail.com (N.V.); neerajbansal341@gmail.com (N.B.); 2 Department of Pharmacology, Pt. B.D. Sharma PGIMS, Rohtak 124001, Haryana, India; shanmugapriyavinayagam@gmail.com (S.V.); drrakeshmittal.pgims@uhsr.ac.in (R.M.)

**Keywords:** prescribing indicators, generic prescribing, rational prescribing, index of rational drug prescribing, polypharmacy, essential drugs

## Abstract

Background: The evaluation of internationally comparable indicators of medicine use is important to devise strategies to promote the rational use of medicines (RUM). Methods: A cross-sectional study was conducted in a tertiary care hospital from January to June 2024. Prescriptions were collected from the outpatient pharmacy using systematic random sampling and analyzed for WHO/INRUD core prescribing indicators, index of rational drug prescribing (IRDP) and completeness (general, treatment and prescribers’ details). Results: Out of 844 prescriptions collected, 607 were analyzed. A total of 1837 drugs were prescribed, with a mean (SD) of 3.03 (1.51) drugs per prescription; 1378 (75%) drugs were prescribed as generic names; 125 prescriptions (20.59%) had an antibiotic prescribed; and injectables were given in 7 (1.15%) prescriptions. Of the total 1837 drugs, 1018 (55.4%) were mentioned in the National List of Essential Medicines (NLEM) 2022, while 934 (50.8%) were included in the Haryana state essential medicines list (2013–2014). The IRDP was calculated as 3.86. The mean (SD) completeness score of the prescriptions was 10.33 (0.8) (range 5 to 11). Conclusions: There was a high incidence of polypharmacy, brand name and non-essential drug prescribing, while antibiotic and injection use were in accordance with WHO standards.

## 1. Introduction

The rational use of medicines (RUM) is crucial in attaining optimal medical and healthcare for patients and the community as a whole. According to the WHO, “Rational use of drugs requires that patients receive medications appropriate to their clinical needs, in doses that meet their own individual requirements for an adequate period of time, at the lowest cost to them and their community” [1]. The irrational prescribing of drugs is a serious global issue. The WHO estimates an approximately more than fifty percent incidence of overuse, underuse and misuse of medicines worldwide, which in turn leads to the wastage of scarce resources and extensive health hazards [1]. Numerous factors through different phases of the medicine use cycle contribute to irrational drug use, some of these being inadequate knowledge and skills among prescribers and patients, prescribing driven by misconceptions, gaps in the drug supply system, weak control and regulations over prescriptions, inappropriate drug promotion strategies by pharmaceutical companies, biased and poor information about medicines, polypharmacy, self-medication, over-the-counter availability of drugs, etc. [2,3]. The irrational use of drugs is linked to many untoward consequences, such as worsening quality and higher cost of drug therapy, increased incidence of undesirable effects (e.g., adverse effects, antimicrobial resistance) and untenable psycho-social impacts on patients [4,5].

A regular and timely evaluation of the rational use of medicines is very essential to take desired actions. In 1993, the WHO, in collaboration with the International Network of Rational Use of Drugs (INRUD), formulated a manual that defines core and complementary drug use indicators for investigating drug use in healthcare facilities at national, regional or local levels in a standardized manner [6,7]; a set of recommended optimum values for each core indicator has also been defined [8]. The core drug use indicators, further divided into prescribing, patient care and facility indicators, help in identifying general prescribing and quality of care problems at healthcare facilities and are recommended for inclusion in drug use studies [6]. Such measures or indicators empower the concerned stakeholders, including healthcare planners, managers and researchers, to compare data across different areas or at different times and take necessary remedial actions [3]. Previous studies from developing countries, including India, have identified significant gaps in prescribing from the WHO/INRUD recommendations [9,10,11,12,13,14,15,16,17,18,19,20,21,22,23,24,25,26,27].

The present study was conducted with an aim of providing insight into the outpatient prescription of drugs in our tertiary care hospital to (1) assess the WHO/INRUD core prescribing indicators, (2) evaluate the completeness of prescriptions, and (3) identify the targets for promoting the rational use of medicines and delivering quality patient care in terms of prescriptions.

## 2. Materials and Methods

### 2.1. Study Design and Setting

This was a cross-sectional observational study conducted by the Department of Pharmacology, Postgraduate Institute of Medical Sciences (PGIMS), Rohtak, Haryana, North India. Located approximately 70 kms from the national capital, New Delhi, our public tertiary care hospital is a major center for the provision of specialized healthcare services not only to the people of the state of Haryana but also to the adjacent states of Punjab, Rajasthan, Delhi and western Uttar Pradesh. The hospital’s outpatient department (OPD), comprising various specialty and super-specialty clinics, witnesses a daily OPD throughput of nearly 10,000–12,000 patients.

### 2.2. Study Objectives

The study objectives included the evaluation of prescriptions for the following:

1. WHO/INRUD core prescribing indicators. These included the following:
  (i). Average number of drugs per prescription/encounter: Total number of drugs prescribed/Number of prescriptions assessed (WHO recommended value: 1.6–1.8);
  (ii). Percentage of drugs prescribed by generic name*:* (Number of drugs prescribed by generic names/Total number of drugs prescribed) × 100 (WHO recommended value: 100%);
  (iii). Percentage of encounters resulting in the prescription of an antibiotic*:* (Number of prescriptions with an antibiotic prescribed/Total number of prescriptions assessed) × 100 (WHO recommended value: 20–26.8%);
  (iv). Percentage of encounters resulting in the prescription of an injection*:* (Number of prescriptions with an injection prescribed/Total number of prescriptions assessed) × 100 (WHO recommended value: 13.4–24.1%);
  (v). Percentage of drugs prescribed from the essential medicines list (EML): (Number of medicines included in EML/Total number of medicines prescribed) × 100 (WHO recommended value: 100%).
2. Index of rational drug prescribing (IRDP).

This is the sum of index values of all prescribing indicators calculated as per the index system developed by Zhang and Zhi for the comprehensive assessment and comparison of different healthcare systems [28].

For indices of non-polypharmacy, antibiotic and safe injection use, the following formula is used:

Index value = (WHO optimal value)/Observed value

The indices of generic prescribing and essential medicines are calculated as

Index value = (Observed value)/WHO optimal value

The optimal values for all the indicators are set as 1, and values close to 1 indicate rational use. Hence, the value of IRDP ranges from 0 to 5.

3. Completeness of prescriptions

This was assessed on the basis of the presence of the following parameters:

- General details: patient demographic details (name, age, sex and address), unique patient ID, date of prescription and diagnosis (4 points);
- Treatment details: name of medicine, dosage form, strength of formulation, dosage regimen/frequency, duration of treatment, and advisory instructions (such as before/after food, at bedtime, etc.) (6 points);
- Prescriber’s signature (1 point).

Each parameter was scored as 1, and prescriptions were allotted a completeness score ranging from 0 to 11 on the basis of the number of parameters present. The percentage of compliance for each parameter was also calculated.

### 2.3. Data Collection and Measures

Paper-based handwritten prescriptions from various clinical departments were collected from the hospital’s OPD pharmacy from January to June 2024. In the OPD pharmacy, there are four separate counters for various groups, such as the general public, females, elderly and medical staff/physically handicapped/policemen. The prescriptions were selected using a systematic random sampling technique. For this, the sampling interval was calculated as the total number of patients in a particular queue (N) divided by the sample of interest (n = 10). A random number between 1 and 10 was chosen to yield the sample start number. For example, if there are 30 patients in a queue (N = 30), the sampling interval is calculated as 3; for a hypothetical random number of 2, the sampling started from the second patient in the queue and proceeded as every third patient subsequently. A similar number of prescriptions were collected from the various counters (mentioned above) so as to ensure the data collected were representative of different groups of patients.

The prescriptions thus collected were screened for potential eligibility in the study. First-encounter prescriptions, i.e., prescriptions of new cases, were included, while prescriptions of follow-up cases and those with illegible or difficult-to-understand handwriting by the study investigators were excluded. From the included prescriptions, information pertinent to the study objectives was extracted using a standardized pre-tested data collection form, viz. date of prescription; demographic details of the patient; unique patient identity number (ID); diagnosis; name of the department; total number of drugs in the prescription; details of the treatment regimen; advisory instructions, if any; total number of fixed-dose combinations (FDCs); names of FDCs; and presence of prescriber’s signatures.

### 2.4. Sample Size Calculation

As per the WHO recommendation, a minimum of 600 prescriptions need to be analyzed to evaluate core prescribing indicators [6]. Hence, a sufficient number of prescriptions were collected in order to have at least 600 prescriptions available for analysis.

### 2.5. Data Analysis

Data were entered into Microsoft Excel and analyzed primarily using descriptive statistics, including frequency distributions (percentages) and means with standard deviations (SDs). No specific hypothesis testing was conducted.

### 2.6. Ethical and Administrative Considerations

The study was conducted after obtaining administrative approval from the office of the Medical Superintendent of the hospital (PGIMS/Misc/23/8944-47) and ethical approval from the Biomedical Research Ethics Committee (vide letter no. BREC/23/497 dated 17 October 2023). Written informed consent was obtained from the patients prior to the collection of prescriptions. Adequate measures were taken to ensure data confidentiality.

## 3. Results

**Figure 1:**
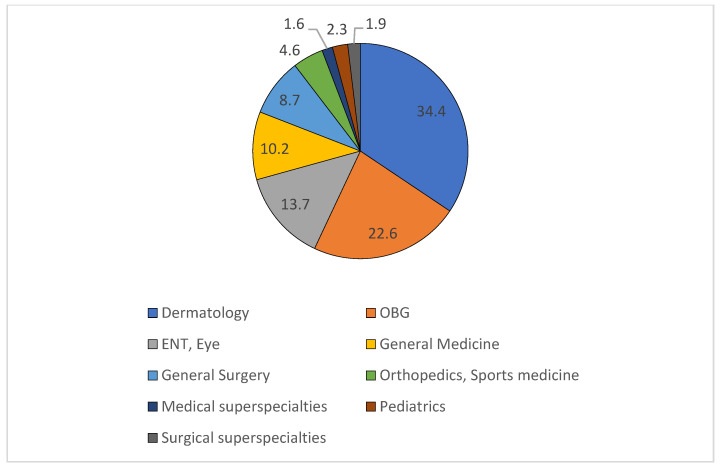
Percentage distribution of prescriptions from various outpatient departments. ENT: ear, nose and throat; OBG: obstetrics and gynecology; medical superspecialties: cardiology, gastroenterology, nephrology, pulmonary and critical care medicine, rheumatology; surgical superspecialties: cardiothoracic surgery, urology, neurosurgery.

Out of 844 prescriptions collected, 220 were follow-up cases, and 17 were illegible; hence, 607 prescriptions were included in the analysis. The prescriptions coming from various specialty and superspecialty departments were collected from outpatient pharmacies for the study (Figure 1).

A total of 1837 drugs were prescribed in 607 prescriptions assessed, with a mean (SD) of 3.03 (1.51) drugs (range: 1 to 9). A total of 93 (15.3%) prescriptions had five or more drugs prescribed. Of the 1837 drugs, 1378 (75%) were prescribed as generic names, and 125 prescriptions (20.59%) had an antibiotic prescribed. Metronidazole (31; 24.8%), amoxicillin-clavulanic acid (29; 23.2%) and cefixime (26; 20.8%) were among the frequently prescribed antibiotics. Injectables were given in seven (1.15%) prescriptions. Regarding essential medicines, of the total 1837 drugs prescribed, 1018 (55.4%) were mentioned in the National List of Essential Medicines (NLEM) 2022, while 934 (50.8%) were included in the Haryana state essential medicines list (2013–2014). A relatively good proportion of drugs were, however, prescribed from the hospital formulary [1596 (86.9%)]. The index of rational drug prescribing (IRDP), calculated by adding all the indices of prescribing indicators, was 3.86 (Table 1).

**Figure 2:**
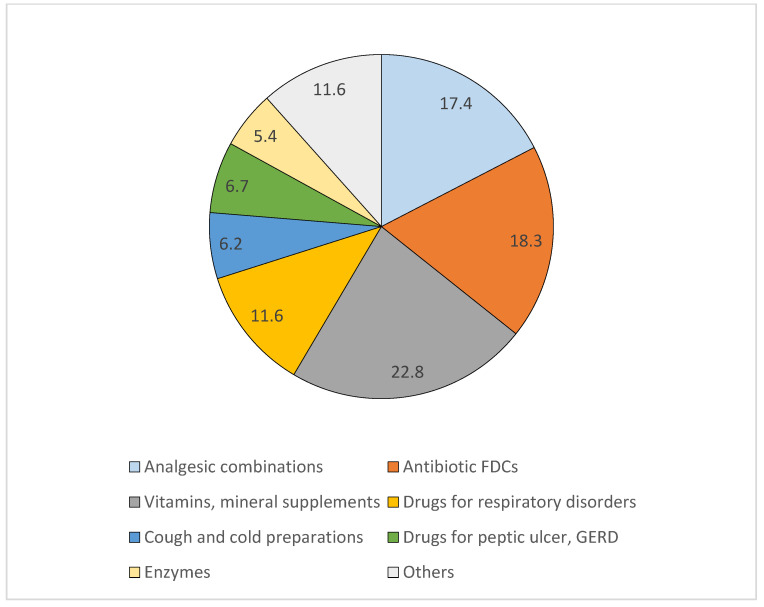
Proportion of various FDCs prescribed. Drugs for respiratory disorders included montelukast + levocetirizine and inhaled beta 2 agonists + corticosteroids; others included FDCs for neuropathic pain, urologic indications such as overactive bladder/benign prostate hyperplasia, cardiovascular indications, combined oral contraceptive pills and iron salt/folic acid. GERD: gastro-esophageal reflux disease.

For the prescriptions, 24.2% (147/607) had at least one FDC prescribed, with a total of 224 FDC prescriptions (12.2% of the total 1837 prescriptions). Vitamins/mineral supplements, nonsteroidal anti-inflammatory drugs (NSAIDs) and antibiotic combinations were among the most commonly prescribed FDCs (Figure 2).

Topical preparations were given in 291 out of a total of 607 prescriptions (47.9%) and mainly included antifungals, anti-acne preparations, antibiotic ear/eye drops and analgesics. The strength of the topical preparation was mentioned in 44.8% (130/291).

### Completeness of Prescriptions

**Figure 3:**
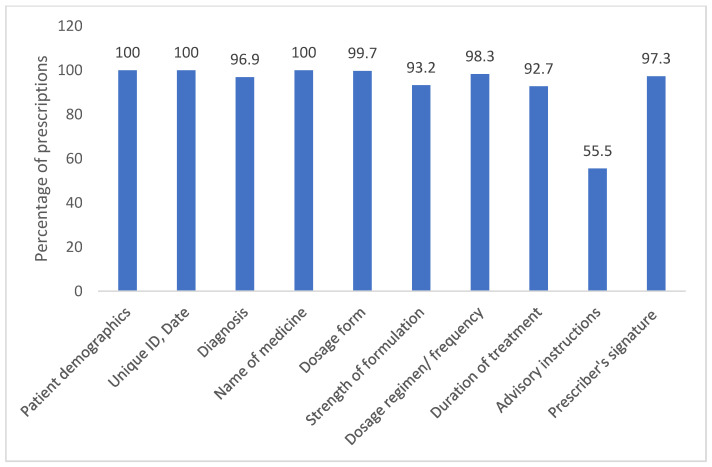
Bar graph illustrating the percentage compliance for each parameter in the outpatient prescriptions analyzed.

The mean (SD) completeness score of the prescriptions was 10.33 (0.8) (range 5 to 11). All the prescriptions mentioned patients’ demographic details and names of medicines. The majority of the prescriptions contained the diagnosis, treatment dosage form and regimen. The strength of formulation and duration of treatment were given in 93.2% and 92.7% of prescriptions, respectively. More than half of the prescriptions mentioned special advisory instructions, and 97.3% of the prescriptions had prescribers’ signatures on them, while 72.16% had a date written by the prescriber (Figure 3). Drug names were written in capital letters in approximately 90% (544) of the prescriptions. None of the prescriptions had the registration number of physicians mentioned in them.

## 4. Discussion

This cross-sectional study analyzed a total of 607 individual outpatient prescriptions, a number based on WHO recommendations and large enough to draw conclusions regarding compliance with WHO/INRUD core prescribing indicators. Data from the study are expected to throw light on prevailing prescription practices in our public tertiary care hospital and define targets to encourage the rational use of medicines in hospitals and the community as a whole. Our findings will also serve as a source of baseline information for the regular monitoring of prescribing practices in our hospital. Similar published studies from other parts of India were also reviewed for comparison purposes. Table 2 provides a comprehensive comparison of the summary findings on WHO indicators from the present study with those from other parts of the country.

An average of 3.03 drugs per encounter were prescribed in our study, with a range of 1 to 9, indicating polypharmacy; this is compared with the WHO-proposed optimal value of 1.6 to 1.8 drugs per encounter. This finding was congruent with drug use patterns in tertiary care hospitals from other parts of India [16,17,18,19,20]. The increasing prevalence of non-communicable diseases with the aging population and associated comorbidities are possible contributing factors towards the prescription of a greater number of medicines per encounter in ours and other tertiary care hospitals in India. Moreover, the majority of the prescribing doctors in such hospitals hold master’s degrees and have been speculated to have a tendency to prescribe a greater number of drugs than their counterparts with bachelor’s degrees [29]. Of note, a few studies from secondary care hospitals also report similar data in this regard, reflecting a widespread prevalence of polypharmacy among outpatient departments of different levels of hospital care in our country [21,22,26]. Besides being associated with adverse consequences, such as decreased adherence, enhanced risk of drug interactions and adverse drug reactions, polypharmacy imposes unnecessary financial burdens on patients as well as the healthcare system. Polypharmacy has also been identified as the single most important predictor of prescription errors; for every additional drug prescribed, the risk of prescription error increases by 14% [30]. Data from other developing and lower-middle-income countries (LMICs) also demonstrate similar prescription patterns on the average number of medicines per encounter [13,14,15,23,24]. In contrast, few studies from Eritrea and Ethiopia report this indicator falling within the frame of WHO standards in outpatient settings [9,10,11,12], which may partly be attributed to variations in study sites and prevailing prescribing practices. Hence, training the prescribers for desired but, at the same time, limited prescribing is a key area of intervention towards achieving the goal of the rational and quality use of medicines. Additionally, the implementation of local evidence-based and optimized policies to curtail polypharmacy is a pressing priority.

WHO clearly enforces the prescribing of drugs by their generic names due to their lower cost and reasonably good accessibility and adherence compared to brand names [6]. Generic drug prescribing was seen in a much greater proportion of prescriptions in our study (75%) when compared to other studies in India, where it ranged from nil [19] to 55.4% [16]. Studies from Pakistan [13], Sri Lanka [14], Tanzania [15] and Kenya [24] also reported poor compliance with the WHO recommended standards of 100% generic name prescribing. Differences in study settings may possibly account for such variation, as generic prescribing is found to be better in public compared to private healthcare settings. A lack of trust among prescribers regarding the quality of generic medicines and trying to prevent pharmacists from dispensing high-cost branded medicines are among other cited reasons for prescribers falling back on brand name prescribing. In this direction, national-level strategies are needed to strongly advocate generic prescribing.

Regarding the encounters with injections, we observed a high degree of conformity with WHO standards, a finding in agreement with studies from other parts of India and other developing countries, which can be explained by the study samples representing outpatient department prescriptions. A lower rate of injections is encouraging, as it helps to avoid unwarranted hazards associated with the use of injectable preparations, such as the increased likelihood of healthcare-associated infections, financial implications, complications due to non-sterile technique, etc. Hence, it is crucial to maintain consistency in the injection use rate with recommended standards by conducting periodic and judicious reviews of prescribing patterns.

In our study, the usage of antibiotics (20.6%) was within the WHO optimal values (20–26.8%) and showed agreement with some other studies from India [16,18,20]. On the contrary, data from studies conducted in some other parts of India and LMICs are alarming, with antibiotic usage exceeding the WHO standards. Irrational antibiotic prescription is a global issue contributing to the emergence and spread of antimicrobial resistance. Adequate measures need to be implemented at regional, national and global levels to promote their optimal use.

In our survey, a relatively lower proportion of the drugs were prescribed from national (55.4%) and state (50.8%) essential medicine lists. There is a huge variation in figures regarding this, as per the published reports from India (ranging from 9 to 100%). Data from other LMICs are, however, contrasting, with most of them resorting to essential medicines. A lack of knowledge of essential medicines may be one reason for this. Limited availability of essential medicines, reported in our earlier survey [31], is another potential factor driving physicians to fall over to non-essential medicines that may be less effective or safe and/or more expensive, leading to poor treatment outcomes and increased healthcare costs [32]. In this direction, a periodic revision of essential medicine lists in line with emerging needs should be emphasized. Additionally, the formulation and implementation of standard treatment guidelines is a key step towards enhancing the quality of prescriptions and achieving RUM. Also, prescribers should be sensitized regarding the importance of essential medicines in optimizing cost-effective prescriptions.

IRPD, as a useful index for district- or region-wide comparisons, may guide policymakers in prioritizing and designing improvement strategies. An IRDP of 3.86 was calculated in our study, which is quite comparable to the reported indices from neighboring LMICs, Sri Lanka (3.58) [14] and Pakistan (3.38 to 4.27) [13]. Some differences in the IRDP across studies from other parts of India (not calculated in any study) may be assumed, considering the values of various WHO indicators in these. Such variations in IRDP may be attributed to numerous factors; for example, the poor penetration and adoption of evidence-based treatment guidelines in comparatively resource-limited areas may translate to greater prescribing of antibiotics and injectables and less generic and essential drug prescribing.

In the present study, 2% (17/844) of prescriptions were illegible, which was less than that reported by other researchers from India (Sunny et al. [20]: 9%, Dhanya et al. [18]: 3.4%, Ahsan et al. [19]: 8.16%). There were inconsistencies in the reporting of completeness parameters for prescriptions across different studies. Patient demographics, date and unique ID were present in all prescriptions, as these are printed on the barcode at the time of registration, which was similar to other studies from India. The diagnosis was, however, mentioned to a lesser extent in some studies (Singh et al. [22]: 64.2%; Shelat et al. [25] 34%; Mulkalwar et al. [17]: 57.2%; Ahsan et al. [19]: 56%) than in ours. The majority of our prescriptions were complete with respect to treatment-related details (name of medicine, dosage form, strength of formulation, dosage regimen/frequency, duration of treatment) compared to a few studies reporting insufficient details in their prescriptions. For example, dosage was mentioned in 10% [25] and 61.2% [16] of the prescriptions in two studies, while incorrect dosages were mentioned in 9% of the prescriptions in the study by Ahsan et al. Duration of treatment was documented in 20% and 87% of the prescriptions in the studies by Shelat et al. [25] and Ahsan et al. [19], respectively. In one study [20], a relatively good proportion of prescriptions (84%) had drug names mentioned in capital letters, which was in alignment with our findings (90%); however, this did not hold true for prescriptions from some other studies (Meenakshi et al. [16]: 17.3%; Mercy et al. [21]: 56.77%; Ahsan et al. [19]: 0%). Very few studies reported registration numbers of physicians mentioned in their prescriptions (Meenakshi et al. [16]: 79.7%; Mercy et al. [21]: 46.77%; Mulkalwar et al. [17]: 88%).

A limitation of the present study is that although the results indicate the areas that are lacking in terms of RUM, the reasons leading to irrational prescribing were not looked upon and revealed. Also, the indicators do not indicate whether the prescribed medicines are in harmony with the diagnosis and standard treatment guidelines. Hence, further studies need to be conducted to explore the reasons for the irrational use of medicines and define improvement strategies accordingly. Another limitation is restricted generalizability to all patients since the data were obtained from different groups of patients having prescriptions from various clinics through systematic random sampling.

## 5. Conclusions

Prescription audit data from the outpatient pharmacy of our tertiary care hospital in North India reflected conformance to WHO optimal values with respect to the number of antibiotics and injections prescribed per prescription. On the contrary, there were deviations from the recommended values for other WHO/INRUD indicators, with a high incidence of polypharmacy, brand name and non-essential drug prescribing. Variable adherence to WHO standards has been observed in previous studies from India, with some reporting a high incidence of antibiotics and injection use as well, which may partly be explained by differences in study settings (public versus private), prescribers’ qualifications and prevailing prescribing practices. Based on our findings and a comprehensive evaluation of data from other parts of the country, the sensitization and periodic training of prescribers on rational drug use is an important target for intervention. The Indian Council of Medical Research (ICMR) has taken a pivotal step in this direction by launching “ICMR- National Virtual Centre Clinical Pharmacology (NvCCP) Prescribing Skills course for Indian Medical Graduates [33]. Further, there is a continuous need to formulate, enforce and periodically revise the standard treatment guidelines. There must be strong political commitments to ensure the availability of essential medicines in a sustainable manner as a key step towards promoting their prescribing. Future studies need to be conducted in different settings to explore the reasons for the irrational use of medicines and define improvement strategies accordingly. Besides these, the periodic sensitization of prescribers toward essential and generic drug prescribing and an emphasis on optimal antimicrobial prescribing are fundamental steps for attaining the goal of rational drug use. Also, the degree of compliance of treatment provided with standard treatment guidelines or protocols needs to be evaluated at various healthcare levels, and any barriers and challenges in adopting the guidelines in routine clinical practice need to be identified and addressed appropriately. 

## Figures and Tables

**Table 1:** Index of rational drug prescribing (IRDP).

S.No.	Index	Values in Study
1.	Index of non-polypharmacy ^a^	0.56
2.	Index of rational antibiotic use ^b^	1.00
3.	Index of safe injection use ^c^	1.00
4.	Index of generic prescribing ^d^	0.75
5.	Index of EML prescribing ^d^	0.55
	IRDP (sum of 1 to 5)	3.86

Optimal value taken as ^a^ 1.7, ^b^ 23.4, ^c^ 18.75, ^d^ 100. Index of EML calculated with respect to NLEM 2022.

**Table 2:** WHO/INRUD core prescribing indicators in studies from different parts of India.

Study, Year of Publication	Setting, Region	No. of Prescriptions Studied	WHO/INRUD Indicators				
			No. of Medicines (Mean)	Injectables (%)	Antibiotics (%)	Generic Prescribing (%)	Essential Medicines (%)
Present study, 2024	Public tertiary care, Haryana	607	3.03	1.15	20.59	75	55.4
Ahsan et al., 2016 [19]	Private, tertiary care, Uttar Pradesh	1274	4.02	7.54	39.01	0	79.2
Meenakshi et al., 2021 [16]	Private, tertiary care, Puducherry	600	2.38	10.5	7.3	55.4	88
Sunny et al., 2019 [20]	Private, tertiary care, Karnataka	500	3.32	3.6	19.4	3.6	9
Mulkalwar et al., 2024 [17]	Private, tertiary care, Pune, Maharashtra	400	3.14	2.25	34	28.72	100
Singh et al., 2019 [22]	Public, secondary care, Delhi	120	3.02	10.8	52.5	85.8	88.3 *
Dhanya et al., 2021 [18]	Public, tertiary care, Kerela	120	3.5	4.8	24.8	45	3.2
Mercy et al., 2022 [21]	Public, secondary care, Puducherry	310	4	39.8	74.12	89.55	94.92 *
Shelat et al., 2015 [25]	Private hospitals, Gujarat	250	3.38	20.8	53.6	6.67	67.54
Potharaju et al., 2011 [26]	Public, secondary care hospitals, Maharashtra	14,004	2.85	25	35	60	46 ^#^
Hazra et al., 2000 [27]	Non-Government organization, West Bengal	312	3.2	3.9	72.8	46.2	45.7 ^#^

* Essential medicine list of hospital. ^#^ WHO Essential Drugs List (EDL) 2003.

## Data Availability

All the relevant data are contained within the article.

## Abbreviations

The following abbreviations are used in this manuscript:

RUM	Rational use of medicines
WHO	World Health Organization
INRUD	International Network of Rational Use of Drugs
NLEM	National List of Essential Medicines
IRDP	Index of rational drug prescribing
OPD	Outpatient department
EML	Essential Medicines List
FDC	Fixed-dose combination
LMICs	Lower-middle-income countries
NvCCP	National Virtual Centre Clinical Pharmacology
ICMR	Indian Council of Medical Research

## References

[1] World Health Organization Promoting Rational Use of Medicines. https://www.who.int/activities/promoting-rational-use-of-medicines.

[2] Ofori-Asenso R., Agyeman A. (2016). Irrational Use of Medicines—A summary of key concepts. Pharmacy.

[3] Mao W., Vu H., Xie Z., Chen W., Tang S. (2015). Systematic review on irrational use of medicines in China and Vietnam. PLoS ONE.

[4] Pan American Health Organization Irrational Use of Medicines. Published 2010. https://www3.paho.org/hq/dmdocuments/2010/3_IrrationalSG.pdf.

[5] Wiedenmayer K., Summers R.S., Mackie C.A., Gous A.G., Everard M., Tromp D. The Pursuit of Responsible Use of Medicines: Sharing and Learning from Country Experiences. ResearchGate. https://www.researchgate.net/publication/269640048_The_pursuit_of_responsible_use_of_medicines_Sharing_and_learning_from_country_experiences.

[6] World Health Organization (1993). How to Investigate Drug Use in Health Facilities: Selected Drug Use Indicators.

[7] World Health Organization (2003). Introduction to Drug Utilization Research.

[8] Isah A., Laing R., Quick J., Mabadeje A., Santoso B., Hogerzeil H., Ross-Degnan D. (2001). The development of reference values for the WHO health facility core prescribing indicators. West. Afr. J. Pharmacol. Drug Res..

[9] Siele S.M., Abdu N., Ghebrehiwet M., Hamed M.R., Tesfamariam E.H. (2022). Drug prescribing and dispensing practices in regional and national referral hospitals of Eritrea: Evaluation with WHO/INRUD core drug use indicators. PLoS ONE.

[10] Amaha N.D., Weldemariam D.G., Abdu N., Tesfamariam E.H. (2019). Prescribing practices using WHO prescribing indicators and factors associated with antibiotic prescribing in six community pharmacies in Asmara, Eritrea: A cross-sectional study. Antimicrob. Resist. Infect. Control..

[11] Gebramariam E.T., Ahmed M. (2019). Evaluation of rational medicine use based on WHO core drug use indicators in public hospitals in West Shoa Zone, Oromia, Ethiopia. Adv. Pharmacoepidemiol. Drug Saf..

[12] Sisay M., Mengistu G., Molla B., Amare F., Gabriel T. (2017). Evaluation of rational drug use based on World Health Organization core drug use indicators in selected public hospitals of eastern Ethiopia: A cross-sectional study. BMC Health Serv. Res..

[13] Atif M., Sarwar M.R., Azeem M., Naz M., Amir S., Nazir K. (2016). Assessment of core drug use indicators using WHO/INRUD methodology at primary healthcare centers in Bahawalpur, Pakistan. BMC Health Serv. Res..

[14] Galappatthy P., Ranasinghe P., Liyanage C.K., Wijayabandara M.S., Mythily S., Jayakody R.L. (2021). WHO/INRUD core drug use indicators and commonly prescribed medicines: A national survey from Sri Lanka. BMC Pharmacol. Toxicol..

[15] Kilipamwambu A., Bwire G.M., Myemba D.T., Njiro B.J., Majigo M.V. (2021). WHO/INRUD core prescribing indicators and antibiotic utilization patterns among primary health care facilities in Ilala district, Tanzania. JAC Antimicrob. Resist..

[16] Meenakshi R., Selvaraj N., Anandabaskar N., Dhamodharan A., Badrinath A.K., Rajamohammad M.A. (2022). Prescription audit of a teaching hospital in South India using World Health Organization core prescribing indicators—A cross-sectional study. Perspect. Clin. Res..

[17] Mulkalwar S., Patel A., David S., Pabari K., Math P., Tilak A.V. (2024). Prescription audit for WHO prescribing indicators and prescription errors in a tertiary care teaching hospital. Med. J. DY Patil. Vidyapeeth.

[18] Dhanya T.H., Sanalkumar K.B., Andrews M.A. (2021). Prescription auditing based on the World Health Organization (WHO) prescribing indicators in outpatient department of a teaching hospital in Kerala. Asian J. Pharm. Clin. Res..

[19] Ahsan M., Shaifali I., Mallick A.K., Singh H.K., Verma S., Shekhar A. (2016). Prescription auditing based on World Health Organization (WHO) prescribing indicators in a teaching hospital in North India. Int. J. Med. Res. Rev..

[20] Sunny D., Roy K., Benny S.S., Mathew D.C., Naik G.J., Gauthaman K. (2019). Prescription audit in an outpatient pharmacy of a tertiary care teaching hospital—A prospective study. J. Young Pharm..

[21] Mercy M., Antony L.J. (2022). A prescription audit using the WHO core drug use indicators in a rural health training center of Pondicherry. CHRISMED J. Health Res..

[22] Singh T., Banerjee B., Garg S., Sharma S. (2019). A prescription audit using the World Health Organization-recommended core drug use indicators in a rural hospital of Delhi. J. Educ. Health Promot..

[23] Galappatthy P., Ranasinghe P., Liyanage C.K., Wijayabandara M., Warapitiya D.S., Jayasekara D., Jayakody R.L. (2021). Core prescribing indicators and the most commonly prescribed medicines in a tertiary health care setting in a developing country. Adv. Pharmacol. Pharm. Sci..

[24] Nyabuti A.O., Okalebo F.A., Guantai E.M. (2020). Examination of WHO/INRUD core drug use indicators at public primary healthcare centers in Kisii County, Kenya. Adv. Pharmacol. Pharm. Sci..

[25] Shelat P.R. (2015). Analysis of outdoor patients’ prescriptions according to World Health Organization (WHO) prescribing indicators among private hospitals in western India. J. Clin. Diagn. Res..

[26] Potharaju H., Kabra S. (2011). Prescription audit of outpatient attendees of secondary level government hospitals in Maharashtra. Indian. J. Pharmacol..

[27] Hazra A., Tripathi S.K., Alam M.S. (2000). Prescribing and dispensing activities at the health facilities of a non-governmental organization. Natl. Med. J. India.

[28] Zhang Y., Zhi M. (1995). Index system, appraising method for comprehensive appraisal. J. North. Jiaotong Univ..

[29] Kun Y. Analysis of Factors Affecting Physicians’ Prescribing Conduct. Published 2002. https://www.semanticscholar.org/paper/Analysis-of-factors-affecting-physicians’-conduct-Kun/ec8dab27292099fc12d919aa772d1a30e0cdaec4.

[30] Seden K., Kirkham J.J., Kennedy T., Lloyd M., James S., Mcmanus A., Ritchings A., Simpson J., Thornton D., Gill A. (2013). Cross-sectional study of prescribing errors in patients admitted to nine hospitals across North West England. BMJ Open.

[31] Mittal N., Mittal R., Singh S., Godara S. (2024). The availability of essential antimicrobials in public and private sector facilities: A cross-sectional survey in a district of North India. Antibiotics.

[32] Shafiq N., Pandey A.K., Malhotra S., Holmes A., Mendelson M., Malpani R., Balasegaram M., Charani E. (2021). Shortage of essential antimicrobials: A major challenge to global health security. BMJ Global Health.

[33] ICMR-SPH. RUD. *ICMR-SPH*. Published 10 August 2022. https://nie.gov.in/icmr_sph/RUD.html.

